# Meetings of Societies

**Published:** 1903-06

**Authors:** 


### MEETINGS OF SOCIETIES.

### Bristol Medico-Chirurgical Society.

*March 11th, 1903.*

Mr. G. MUNRO SMITH, President, in the Chair.

Dr. J. ODERY SYMES showed **microscopical specimens of the blood obtained from a case of quartan malaria** in Cyprus.

Dr Newman Neild showed a specimen of **tumour of the pituitary body.** At the *post-mortem* the head had been opened first. The meninges were normal. The convolutions of the cerebral cortex were rather flattened. The tumour, the size of a walnut, was seen on raising the frontal lobes. The greater portion of it lay above, and a small portion within, the sella turcica. The latter was considerably enlarged. The posterior clinoid processes were displaced backwards and slightly outwards, and the left cavernous sinus pressed upon. The tumour had grown backwards beneath the pons and forward beneath the cerebral peduncles and had caused an abnormal separation of the carotid arteries. No trace of the pedicle of the pituitary body was found, although a complete segittal section of the brain had been made passing through the apex of the infundibulum. The substance of the tumour was softer than brain tissue and hemorrhagic in parts, the surface being minutely mammillated. The other organs of the body were normal, the thyroid being especially examined. There were no signs of acromegaly. Microscopically, sections at first sight resembled carcinoma or sarcoma, but appeared to be adenoma. The tumour showed no tendency to invade surrounding tissues. A small portion from within the sella turcica, the size of a small pea, showed a few small cysts containing colloid material. Dr. Symes stated that the patient from whom the above specimen had been obtained, was a child of seven years of age. The child had been well until August 4th. Then pain in the head and left eyebrow had developed, and the child had become drowsy and irritable, and had had occasional vomiting. Then ptosis of the left upper eyelid had become noticeable, and, later, the external rectus muscle of that eye became paralysed. There was no optic neuritis. The pulse had been usually slow. On October 17th the urine had contained much albumen. Complete left ocular paralysis then developed. On October 7th the patient had died suddenly. Absence of optic neuritis had been noted in other cases of the kind. The tumour was larger on the left side than on the right. The left knee-jerk had been exaggerated.—Dr. Newman Neild suggested that the spreading of the tumour backwards over the pons caused the ocular paralysis, rather than pressure on the cavernous sinus.—Dr. Shingleton Smith stated that a steatoma of the pituitary body had been shown to the Society some years previously. Symptoms had only been manifest shortly before death, although the tumour was probably of many years growth.—Mr. Roger Williams alluded to Marie's discovery of a relationship between pituitary heterotrophic enlargements and acromegaly. It was curious to find a tendency to the production of thyroid-like tumours at each end of the notochord. Pathologists had too readily adopted the view that certain heterotrophic tumours were teratomata. In the pituitary region such tumours were due to the up-growth of the pouch of Rathké through the tissues forming the base of the skull. With this up-growth matricular elements were carried and accounted for the heterotrophic elements found in the tumours. Brestlau and Rindfleisch had reported such a tumour which had one segment projecting from the mouth and another into the cranial cavity. It had a cystic structure and contained bone, etc., and had been said to contain intestinal elements, and to be of a fœtal nature. It had more probably originated in the way just described.—Dr. Theodore Fisher referred to a paper by Dr. Lloyd Andriezen, in which evidence was brought forward to show that the posterior half of the pituitary body produces an internal secretion, which plays an important part in the nutrition of the central nervous system. A few cases the speaker had seen recorded did not seem to lend support to this view, but there had been one case at the Bristol Royal Infirmary, in which death had occurred in the way described by Andriezen, and the only lesion found after death had been a gumma of the pituitary body invading the floor of the third ventricle.

Dr. Bertram Rogers showed the **liver from a case of lymphadenoma.** The glandular enlargements in various parts had been very rapid. Those in the neck had become enormous. Suppuration occurred in one gland, and subsequently there had been a diminution in size of all the other glands.—Dr. George Parker said that the diminution in the size of the glands which occurred in these cases was only temporary. He mentioned a case in which the blood became normal once or twice a year.

Mr. G. Munro Smith reported a case of **perforated duodenal ulcer** in a man of 33, for which he had successfully operated. The man had had dyspepsia for many years. The pain generally occurred two or three hours after meals and also during the night. One night, at eleven o'clock, he was seized with violent pains in the right side of the abdomen, for which he came to the Infirmary. The diagnosis rested between perforation of a duodenal ulcer and appendicitis. After a few hours the abdomen was opened, first in the lower part revealing a normal appendix, and then above exposing a perforation of a duodenal ulcer. This was closed with Lembert sutures and the peritoneum around mopped out, a gauze drain used above and a tube passed through the lower wound. Sixteen days later the patient had had an attack of intense abdominal pain, but it had soon passed off. The symptoms of duodenal ulcer which the patient had had were fairly typical. These ulcers occurred three times more frequently in men than in women. Not many cases of operation for these ulcers had been published. The first successful operation had been by Dean, in 1894. So far no symptoms of cicatricial contraction had arisen in the case described.—Dr. Shingleton Smith thought it would have been very difficult to diagnose duodenal ulcer from the symptoms which had been mentioned, and asked if there had been any history of hemorrhage from the bowel. He regarded such hemorrhage as often suggestive of the affection.—Mr. LACE said that in eleven cases of perforated gastric ulcer he had examined there had been invariably tenderness in the lower part of the abdomen. Also he had invariably noticed that after an hour or two the temperature had been raised a degree or two. In all the cases he had seen operated upon the abdomen was irrigated and apparently always with benefit, for all the eleven cases had recovered. Seven of these had been his own cases. Drainage for long periods was not necessary.

Dr. E. W. H. GROVES read a paper on **some points in the diagnosis of malignant disease of the body of the uterus,** and showed specimens.—[For this paper see p. 128].—Mr. ROGER WILLIAMS maintained that no pathologist could say from mere histological appearances of scrapings or small bits of tissue whether a uterine condition was malignant or not. In forty cases of uterine cancer one would be of the body of the uterus. Cancer of the body of the uterus was a senile affection occurring from the age of 55 upwards. It was hardly ever met with before the menopause. He considered that the name adenoma was wrongly given to some uterine changes which were hyperplastic and reparative in nature.—Dr. WALTER SWAYNE considered the treatment which Dr. Groves had adopted had been perfectly correct. In his view, in any case in which after curetting a suspicion of malignant disease was raised by microscopical examination, and a rapid recurrence of the clinical symptoms occurred, the case should be looked upon as one of malignant disease. He mentioned several cases of his own, especially two, in which malignant disease developed in the uteri of patients already affected with fibroids. In both these the clinical symptoms recurred almost at once, whereas in a uterus with fibroids, where curetting had been done for the relief of hemorrhage, the symptoms were relieved, sometimes for two or three years. He regarded the early onset of pain in these cases as a most important feature. He was accustomed to place reliance for diagnostic purposes more on clinical symptoms and physical signs than on microscopical evidence, even if the latter was negative. He described the case of a woman, aged 57, whose uterus he explored and curetted for hemorrhage; benign adenoma or hyperplasia of the endometrium was found. This was evidenced by the naked eye appearance and confirmed by the microscope. The hemorrhage recurred within six weeks, and three months later he had found on a second exploration that the growth was more marked than before and left a ragged surface on removal. He had removed the uterus, which proved to be the seat of malignant disease. He considered that the complication with fibroids and polypus added greatly to the difficulties of diagnosis in Dr. Groves's case, and mentioned a case of his own in which, after many operations for recurrent polypus, the uterus was found to be the seat of sarcoma. He alluded to senile atrophic endometritis as being easily mistaken for malignant disease; but the important point to remember was that these simple affections, as a general rule, were cured by curetting, at any rate for a considerable time; malignant disease, on the other hand, rapidly recurs, even if relief was obtained for a few weeks.—Dr. THEODORE FISHER referred to the pain which had been mentioned in connection with carcinoma of the uterus, and also to the muscular hypertrophy which had been described. He said that the amount of hypertrophy of the muscular tissue of hollow viscera which was produced by the slower growing forms of carcinoma was very remarkable. At one time he thought the hypertrophy to be probably due to muscular contractions set up with a view to the expulsion of the growth. Having, however, seen very great muscular hypertrophy set up by a secondary growth in the diaphragm, he now thought that theory of the causation of the increase of muscular tissue improbable. But although the explanation of the hypertrophy was obscure, he thought that the pain of carcinomata in hollow viscera was probably largely due to contractions of this muscular tissue during its growth.

---

*April 8th, 1903.*

Mr. G. MUNRO SMITH, President, in the Chair.

Dr. WALTER C. SWAYNE showed a **placenta prævia with velamentous insertion of the cord.** The case from which the specimen was obtained had been one of complete placenta prævia, and had been treated by dilatation of the os and version. When the os was being dilated a small portion of placental tissue came away, and this on subsequent examination was found to be a succenturiate placenta. The association of a placenta prævia with velamentous insertion of the cord and the existence of a placenta succenturiata was remarkable.

Mr. H. F. MOLE read notes on **two unusual cases of death from being run over.** The first case was that of a boy of 16, who had been run over as a result of a bicycle accident, and the wheel of the vehicle which inflicted the injury appeared to have passed over the right shoulder and upper part of the chest. Emphysema was very marked, so that the ribs could not be felt, and the emphysema could be seen to be rapidly spreading to the head and other parts. Cyanosis increased quickly, and death rapidly followed. At the autopsy six ribs were found to have been broken, but the parietal pleura was not torn. There was a small rent of the anterior border of the right lung. **The right bronchus had been completely torn across** and separated from the lung. In the second case the injury was chiefly in the right groin, and a peculiar bluish-coloured swelling rapidly formed precisely in the position where usually the swelling is seen in a case of extravasation of the urine in the scrotal, pubic and iliac regions. Anæmia soon became profound, and death was rapid. At the autopsy **the external iliac artery was found completely torn across** and also the abdominal muscles overlying it.—Mr. ROGER WILLIAMS alluded to a case in which artificial respiration had been used for a man who had ceased breathing under chloroform. The patient rallied, but subsequently had hæmoptysis, and in two days died. The autopsy showed that a lung and even the pericardium, had been ruptured by the violent pressure on the thorax during the performance of the artificial respiration. Another peculiar chest injury he had seen was that of a patient who had been stabbed in the heart region, and the heart had been wounded without the pericardium being punctured; apparently the knife had been ensheathed by the pericardium when the heart was wounded. The patient died in a few days from violent hemorrhage from the internal mammary artery which had also been wounded.

Dr. A. B. PROWSE read notes on a case of **sudden death in a child from a rare cause**, and showed a specimen obtained from the case. The patient, a lad of five years of age, had been admitted to the Bristol Royal Infirmary, suffering from somewhat indefinite symptoms. He had had a fit before admission. He had no marked dyspnœa. The tonsils were rather large. After a week or two some stiffness of the neck developed, and some of the cervical glands enlarged in the upper part of the neck. One day, suddenly and without warning, blood poured out from the mouth and nose. Tracheotomy was performed as suffocation seemed imminent, but in a few minutes the patient died. A small abscess was found *post-mortem* in one side of the neck near the lateral wall of the pharynx, and this had opened into the pharynx. There was also a small ruptured aneurysm of the internal carotid artery just before it passed into the temporal bone; apparently the aneurism had arisen from softening of the arterial wall through the suppuration in its neighbourhood. No other vascular lesions had been discovered.

Dr. W. C. SWAYNE read notes on a case (*a*) of **ovarian tumour**, and (*b*) of **uterine fibroid**. The first case was that of a nulliparous woman of 43 years of age. Three years previously the speaker had removed for her a large ovarian cyst by an operation which had proved extremely difficult owing to the presence of numerous firm adhesions. Last November he had operated again to remove a similar cyst of the other ovary. The second operation had proved even more difficult than the first. The second case was that of a nulliparous woman of 29 years of age. For two years she had suffered from painful and excessive menstruation and for five months had noticed a swelling in the hypogastrium. The tumour was evidently uterine, and together with that organ was about the size of the pregnant uterus at four months. Panhysterectomy was performed. The vaginal mucous membrane around the uterus had first been divided and then the abdomen opened. The myoma screw failed to raise the uterus and tumour out of the pelvis until the peritoneal covering had been stripped off very extensively. The difficulty in getting the tumour out was due to the fact that both the Fallopian tubes were dilated and embedded in exudation and had anchored the tumour in the pelvis. There had been much collapse at the end of the operation, with a rapid pulse. Saline fluid was poured into the abdomen and this lowered the pulse rate. At the end of thirty hours the patient's condition became very bad again. Saline fluid was again poured into the abdomen after removing a stitch and passing in a tube. At the end of the second week there was a rise of temperature, which was found to be due to a collection of pus retained in the uppermost part of the vagina by adhesions between the anterior and posterior vaginal walls. Recovery ensued.—Mr. ROGER WILLIAMS stated that patients had become pregnant after the removal of such tumours on both sides. Many authorities had denied that supernumerary ovaries were ever found, but there appeared to be a few cases where their existence had been well authenticated ; but the existence of accessory nodules of ovarian tissue in neighbouring parts in addition to the ovaries proper was more frequent, and possibly explained the recurrence of ovarian tumours or the occurrence of pregnancy after the removal of both ovaries.—Dr. E. H. STACK said that the last speaker's remarks reminded him of a case in which he had seen Macan operate for an abdominal swelling which had formed subsequently to the removal of both ovaries, and the tumour had proved to be due to extra-uterine pregnancy.—Mr. MUNRO SMITH asked why the hysterectomy had been begun from the vagina.—Dr. SWAYNE replied that many cases of panhysterectomy had done badly because the vagina had not been thoroughly disinfected. To effect such disinfection, the vagina had to be well swabbed with antiseptics. Having completed that proceeding in his case, and the patient being in a suitable position, he had made his first incision in the vagina. He remembered a case in which he had removed both ovaries when performing hysteropexy; subsequently the patient had regained sexual power previously lost, and had consulted him for a swelling which felt very like an ovary.

Mr. A. W. PRICHARD read notes on **three cases of perforated gastric ulcer**, in each of which he had successfully operated. The first case had been operated upon about five hours after signs of perforation. A supra-pubic drainage-tube was used and the abdomen swabbed ; by the nineteenth day the patient was well. In the second case there had been pain in the right side for two days and previous indigestion. It was not possible to say exactly when the perforation had occurred. To effect closure of the perforation the whole ulcer had had to be inverted into the stomach. Four drainage-tubes had been used, one on each flank, one above the pubes, and one through the main abdominal incision. The third case, a factory girl, aged 20, had been operated upon forty-eight hours after the onset of pain, with a distended abdomen and a pulse of 180. No one who saw the patient thought she could recover. One tube was passed from the left flank to the main incision, and another through the vagina. Saline fluid was poured into the abdomen.—Mr. R. G. P. Lansdown alluded to the heated discussions which had taken place with respect to the question of irrigating the peritoneal cavity in these cases. He was of opinion that no hard and fast rule could be made; that in some cases irrigation was of value, in some it would be criminal. The advantage of making the main incision rather to one side of the middle line than accurately in the middle line was referred to.—Mr. J. Lacy Firth thought it an interesting point in the second case that it had been impossible to say from the history when the perforation had occurred. In a certain proportion of cases the symptoms at the time of perforation were not definite. He alluded to a case in which he had operated on a patient after an illness of two or three weeks, and evacuated about a couple of pints of pus which had collected between the parietal peritoneum in front and the great omentum behind, and was secondary to a perforated gastric ulcer. In two other cases he had operated for symptoms of gastric perforation, and found the perforations in these ulcers had become sealed up though there was lymph and turbid fluid in the region around. Both the cases had recovered.—Mr. Lace stated that he and his colleagues had operated on twelve cases of perforated gastric ulcer: one only of the cases had not been successful. In all the abdominal cavity had been as thoroughly irrigated as was possible.—Dr. Stack said that the most important point of the technique was free drainage and not irrigation or swabbing. The cases did best which were most freely drained.—Mr. Paul Bush pointed out that at the time the outcry against irrigation was first raised it was customary to use strong antiseptics for the purpose. The use of sterile non-irritating fluid for the purpose rather altered the aspect of the question.—Mr. Mole remarked that some cases did well with mopping out only, others did as well after irrigation. Washing out was good for cases where the operation was late, or the stomach had been full at the time of operation. He considered that drainage above the pubes or through the vagina was important, and also through the left flank. In fatal cases he had seen several times a collection of pus in the left flank.—Mr. Prichard thought that swabbing out only was better for cases of peritonitis from intestinal lesions.

*May 20th, 1903.*

Mr. G. MUNRO SMITH, President, in the Chair.

JOHN CHIENE, C.B., LL.D., F.R.S.E., M.D., Professor of Surgery, University of Edinburgh, gave an address on Mobility. *Vide* page 97.

The PRESIDENT proposed, Mr. N. C. DOBSON seconded, and it was carried with acclamation: "That the best thanks of the Society be accorded to Professor CHIENE for his able and entertaining address."

J. LACY FIRTH.
J. PAUL BUSH (*Hon. Sec.*).

